# An Improbable Thromboembolic Manifestation of COVID-19: A Case Report

**DOI:** 10.7759/cureus.23013

**Published:** 2022-03-09

**Authors:** Fátima Costa, Luís Nogueira, Salomé Marques, Liliana Torres, Ana Filipa Silva

**Affiliations:** 1 Internal Medicine, Centro Hospitalar Tâmega e Sousa, Penafiel, PRT

**Keywords:** thromboembolism, abdominal pain, mesenteric ischemia, emergencies, covid-19

## Abstract

The coronavirus disease 2019 (COVID-19) disease is a multisystem disease and recent studies have shown an increase in reported thromboembolic complications as deep venous thrombosis, pulmonary embolism (PE), stroke, and less frequently mesenteric artery thrombosis. We present a case of a 75-year-old woman, COVID-19 positive with five days of evolution, who was admitted to the emergency room due to diffuse abdominal pain with several days of progression, along with diarrhea and biliary vomit. Abdominal computed tomography presented images of subtraction of the lumen of the upper mesenteric artery. With the reported clinical case the authors intend to clarify the importance of differential diagnosis in patients with a typical severe acute respiratory syndrome coronavirus 2 (SARS CoV2) infection presentation. The gastrointestinal symptoms of SARS CoV2 infection can mask a more severe condition, so a high index suspicion for abdominal thromboembolic events is required once this complication may threaten patient’s life.

## Introduction

The coronavirus disease 2019 (COVID-19) has multiple forms of presentation. Although it mainly affects the respiratory system, in the form of pneumonia, it can target almost every system, such as the gastrointestinal [1] and neurologic, and more recent studies have shown an increase in reported thromboembolic complications [2]. It is known that the membrane of the virus has a spike protein [3], which binds to the angiotensin-converting enzyme 2 (ACE2) receptor expressed in vascular endothelium. The expression of ACE2 on enterocytes of the small bowel may result in intestinal tropism and damage. Additionally, the binding of severe acute respiratory syndrome coronavirus 2 (SARS CoV 2) to ACE2 reduces the degradation of angiotensin II, and consequently stimulates the production of interleukin 6, which induces the production of tissue factor and plasminogen activator inhibitor-1, leading to a hypercoagulable state [4]. Thereby, the mechanisms behind thrombotic complications are coagulation disorder induced by a systemic inflammatory state, endothelial activation, hypoxia, and immobilization [4].

Along with deep venous thrombosis, pulmonary embolism (PE), and stroke, acute mesenteric ischemia (AMI) has been reported in severe COVID-19 patients. AMI is a severe complication with a high mortality rate. The risk is increased in patients with advanced age, cardiac arrhythmias, cardiac valvular disease, infective endocarditis, recent myocardial infarction, ventricular aneurysm, aortic aneurysm or atherosclerosis, peripheral artery disease, and low cardiac output states [1]. Early symptoms and clinical signs are nonspecific, but the most frequent form of presentation is acute-onset abdominal pain along with minimal findings on abdominal examination, classically described as pain disproportionate to physical examination findings. If metabolic acidosis is concomitantly present, intestinal ischemia should be considered until proven otherwise. The diagnosis is based on high-resolution computed tomographic (CT) angiography showing the occlusion within the mesenteric arteries. The definitive treatment consists in vascular intervention to restore intestinal blood flow [5].

## Case presentation

A 75-year-old woman, with a clinical frailty score of 7, COVID 19 positive with five days of evolution, was admitted to the emergency room (ER) due to abdominal pain with several days of evolution, initially localized in the hypogastric region, but widespread at admission, along with diarrhea and biliary vomit. The results of the analytical investigation are in Table 1. 

**Table 1:** Laboratory results

Parameter	Result	Reference value
Leucocytes	20,770/µL	4,500-11,000/µL
Neutrophils	86.70%	55-70%
Lymphocytes	4.40%	20-40%
C-reactive protein	130.5 mg/L	<5.0 mg/L
Procalcitonin	5.66 ng/mL	<0.5 ng/mL
D-dimer	3,599 ng/mL	<243 ng/mL
Lactate	3.2 mmol/L	<2 mmol/L
Potassium	3.1 mmol/L	3.5-5.1 mmol/L
Alanine transaminase (ALT)	173 UI/L	10-31 UI/L
Aspartate transaminase (AST)	39 UI/L	10-31 UI/L
Alkaline phosphatase (ALP)	168 UI/L	30-120 UI/L
Gamma-glutamyl transpeptidase (GGT)	273 UI/L	7-32 UI/L

As we can see, there was increased systemic inflammatory markers, elevated D-dimer, hyperlactacidemia, hypokalaemia, and increased liver and cholestasis enzymes. Bilirubin was normal and initially there was no hypoxemia (pO_2_ 76 mmHg).

Physical examination revealed a tympanitic abdomen, diffusely painful to percussion and superficial palpation, with evidence of peritoneal irritation.

Past medical history included arterial hypertension, diabetes mellitus, dyslipidemia, ischemic heart disease, and atrial fibrillation. She was taking ramipril 10 mg id, metformin 500 mg bid, sinvastatin 20 mg id, furosemide 40 mg id, bisoprolol 5 mg id, apixaban 5 mg bid, and alprazolam 0.5 mg id.

**Figure 1:**
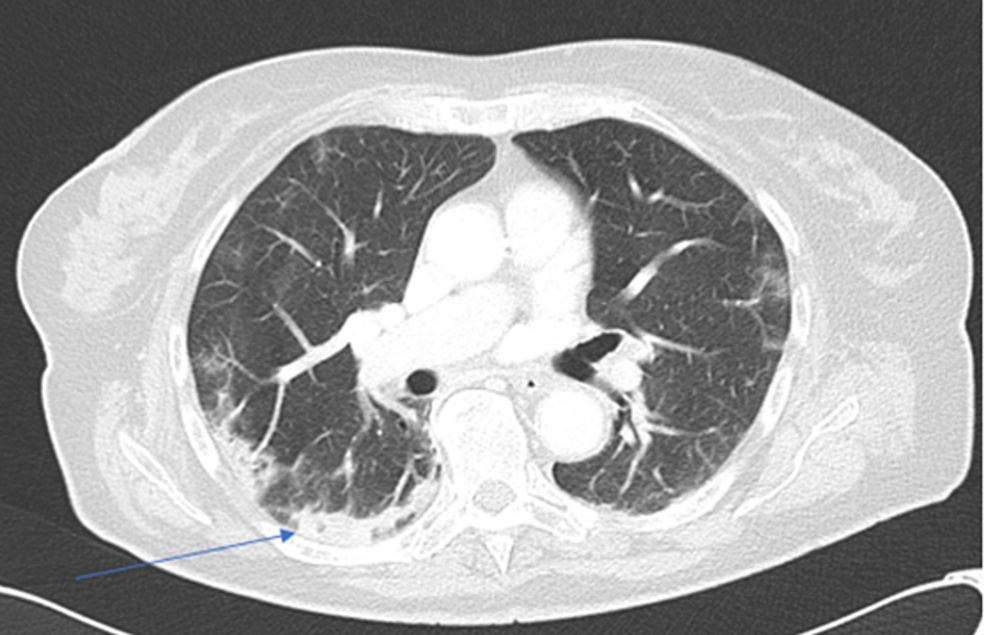
Thoracic CT showing areas of organizing pneumonia (arrow) CT, computed tomography

**Figure 2:**
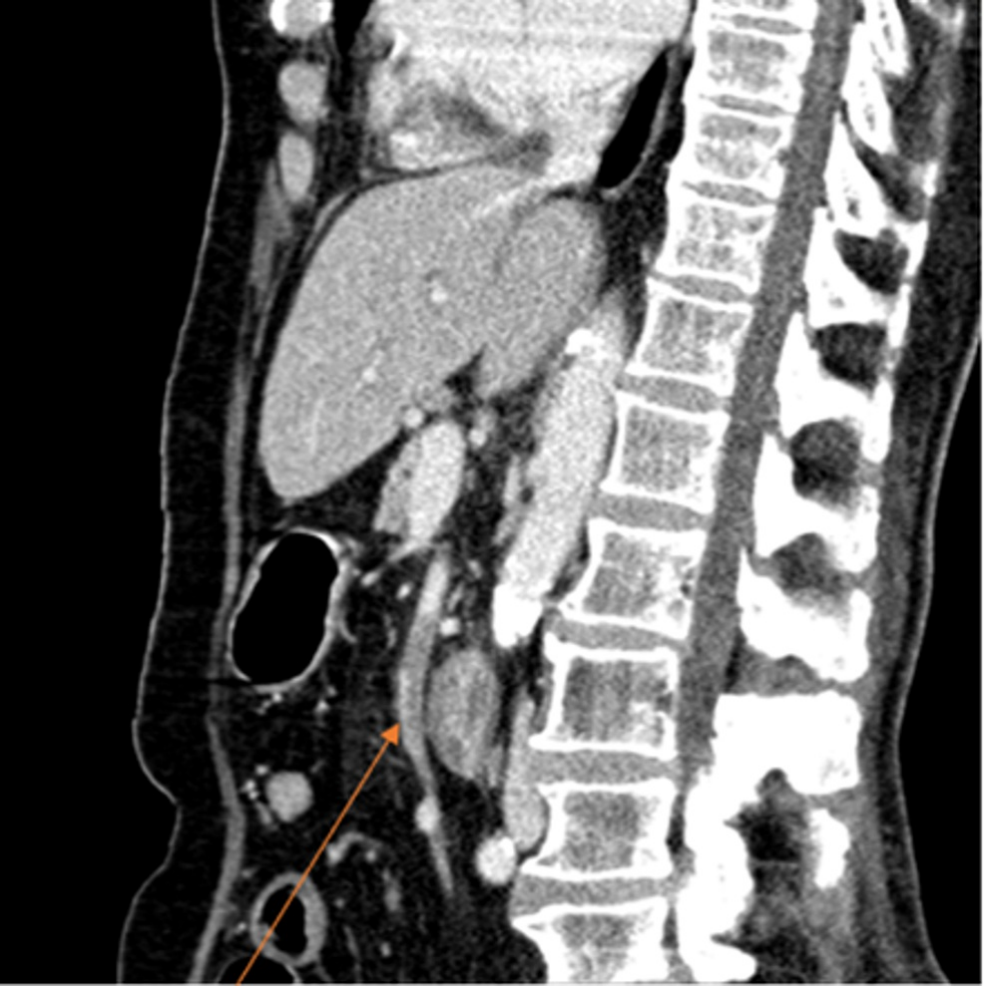
Abdominal and pelvic CT showing subtraction of the lumen of the upper mesenteric artery (arrow) CT, computed tomography

Thoracic, abdominal, and pelvic CTs were performed and revealed multiple densifications, predominantly peripheral, in the parenchyma of both lungs; "ground glass" that were consistent with mild COVID-19 manifestation and some areas of organizing pneumonia (Figure 1); normal-sized liver; gallbladder without calcified lithiasis or acute cholecystitis; ectasia of the main biliary tract that reveals 13 mm of caliber without evidence of hyperdense foci in its lumen; and images of subtraction of the lumen of the upper mesenteric artery were found (Figure 2), translating thrombosis phenomena in a significant part of its path, some distension of loops in the small intestine with normal permeability of the superior mesenteric vein and its branches.

Given the findings, exploratory laparotomy was performed. There was irreversible mesenteric ischemia, so it was decided to privilege conservative treatment with therapeutic enoxaparin (1 mg/kg bid) and comfort measures with morphine and ropivacaine (for five days).

At day 10, a new abdominal pelvic CT was performed and re-permeabilization of the lumen of the superior mesenteric artery was observed, with no thrombi, along with a normal permeability of the celiac trunk and the inferior mesenteric artery. There was also dilation of several loops of the small intestine, filled with liquid, with a thin wall, without relevant highlight, aspects that were consistent with previous ischemia.

Favorable evolution was verified, with improvement of the inflammatory parameters, as well as the clinical condition, so it was decided to perform a new laparotomy at day 16. New laparotomy findings consisted of small intestine gangrene and generalized enteric peritonitis, and it was decided to maintain the initial strategy. There was a clinical decline, with a worsening of the state of dependence and she was admitted to a palliative care unit.

## Discussion

COVID-19 is a multisystem disease with multiple forms of presentation, and the gastrointestinal tract is one of the most affected system (17.6% of patients with SARS CoV2 infection [6]), after the respiratory tract. The most common gastrointestinal symptoms are anorexia, nausea, vomiting, abdominal pain, and diarrhea. It can be explained by the high expression of ACE-2 receptor in the epithelial cells of the bowel. Gastrointestinal symptoms may develop in the early stages of the disease, even before respiratory manifestations; the stomach ache in the usual gastroenteritis is generally less stronger than in mesenteric ischemia and is not associated with peritoneal irritation as in this patient. Apart from gastrointestinal signs, COVID-19 patients often present with elevated alanine transaminase and aspartate transaminase levels, which can be explained by the higher levels of ACE-2 expression in cholangiocytes, with cholangiocyte dysfunction causing liver damage [7]. Additionally, the systemic inflammatory response and stress, as well as hypoxia that causes ischemia, can damage the hepatocytes. 

Another manifestation of COVID-19 is thromboembolic complications. Alongside deep venous thrombosis, PE, and stroke, mesenteric artery thrombosis should be considered. The virus rises the incidence of mesenteric ischemia from 0.09% to 0.2% in the normal population to 1.9% to 3.8% [8,9]. 

## Conclusions

In this case we have an old woman, hypocoagulated, with typical gastrointestinal symptoms of COVID-19. Given her hypocoagulation therapy, a thrombotic phenomenon of this extension would not be expected. For this matter, the authors enhance the need for a high-index suspicion in order to identify abdominal thromboembolic events, as this is a life-threatening situation. Also, this clinical case highlights that gastrointestinal symptoms of SARS CoV2 infection can mask a more severe condition.
